# Supercharged Free Transverse Rectus Abdominis Myocutaneous Flap: An Autologous Reconstructive Option for the Thin Breast Reconstruction Patient

**DOI:** 10.7759/cureus.8776

**Published:** 2020-06-22

**Authors:** Gene Lee, Austin J Pourmoussa, David Perrault, Alex K Wong

**Affiliations:** 1 Plastic Surgery, Keck School of Medicine of the University of Southern California, Los Angeles, USA

**Keywords:** microvascular free flap, breast reconstruction, supercharged flap, microsurgery, breast cancer, tram flap

## Abstract

The free transverse rectus abdominis myocutaneous (fTRAM) flap is a frequently used option for autologous breast reconstruction, typically based on deep inferior epigastric vessels anastomosed to either the axillary or internal mammary systems. The distal portion of the fTRAM flap is routinely discarded prior to anastomosis, due to tenuous blood supply in the vascular territory most distal to the pedicle. This becomes problematic in cases that require use of the entire flap, such as in thin patients with large soft-tissue defects. We report a case where an additional “supercharged” venous microsurgical anastomosis was successfully performed to minimize adverse events while utilizing the entire fTRAM flap.

## Introduction

Autologous breast reconstruction with the pedicled, free, and muscle-sparing transverse rectus abdominis myocutaneous (TRAM) flap has become a frequently used reconstructive option throughout its evolution, owing to its outstanding aesthetic outcome without the need for prosthesis, superior long-term result, and high patient satisfaction. The free TRAM (fTRAM) flap has traditionally been based on the deep inferior epigastric vessels anastomosed to either the axillary or internal mammary systems; however, this technique has been plagued with varying degrees of complications related to the unpredictable flap blood supply, including skin necrosis, fat necrosis, and partial and total flap loss [1]. We present in this report the unique method of augmenting the vascular territory of zone 4 of the fTRAM flap with an additional “supercharged” venous microsurgical anastomosis. 

## Case presentation

A 47-year-old female, with a history of left breast cancer and total mastectomy followed by neo-adjuvant radiation in 2009, presented to the Plastic and Reconstructive Surgery clinic in December 2014 to discuss options for breast reconstruction. Other significant past medical and surgical histories at the time of her initial presentation included a left posterolateral thoracotomy for complicated pneumococcal pneumonia, three cesarean sections, and 1 ppd tobacco use. Her disease status was closely monitored by her oncologic team and was deemed disease-free as evident in her then most up-to-date negative screening mammogram in 2013. 

**Figure 1:**
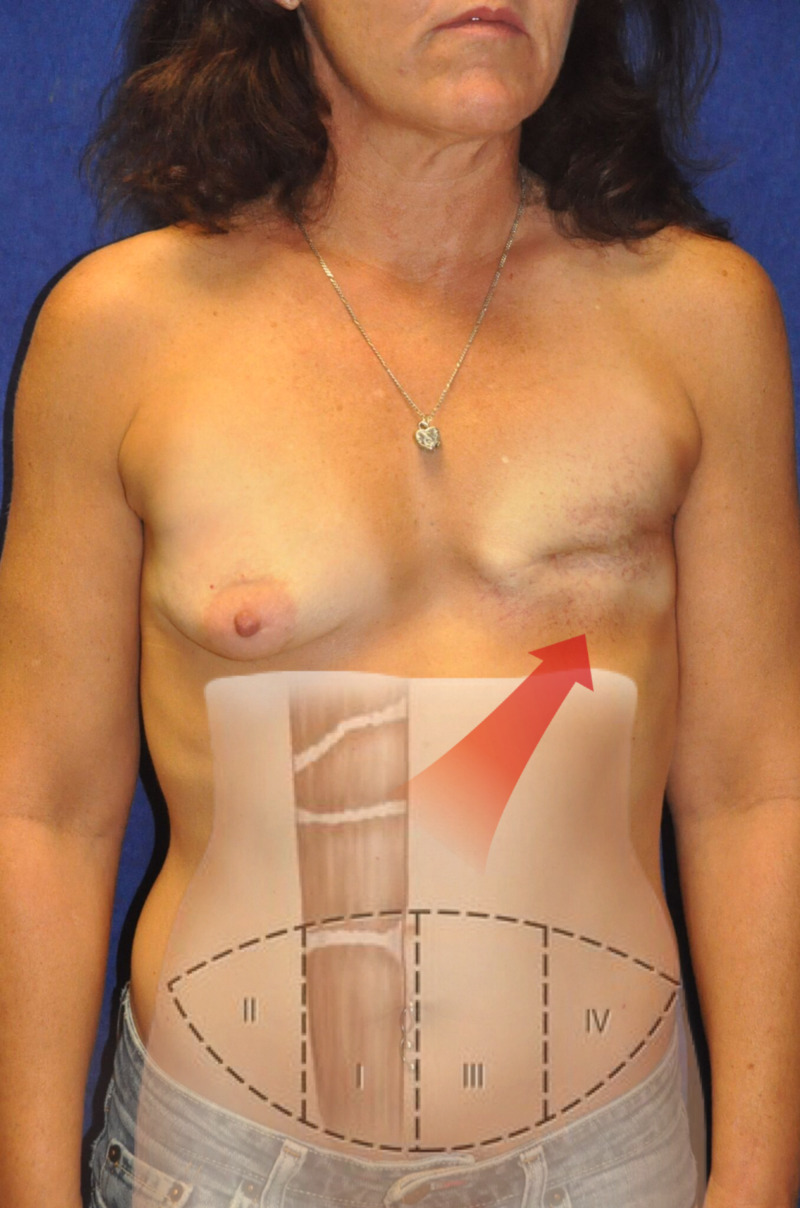
Preoperative photo of the patient, superimposed with an outline of the Holm TRAM flap vascular zone classification. Zone I overlies the area supplied by the dominant pedicle being used for the microsurgical anastomosis. Zone II lies lateral to zone I on the ipsilateral side. Zone III lies across the midline adjacent to zone I. Zone IV lies lateral to zone III on the contralateral side of the pedicle. TRAM, transverse rectus abdominis myocutaneous.

Upon successful cessation of her tobacco use for a period of six months after the initial consultation, the patient was taken to the operating room in June 2015. With goals to recreate a full C-cup sized breast in a patient with a very thin body habitus (body mass index = 24), our team utilized a supercharged muscle-sparing fTRAM flap in order to safely utilize all four zones of the abdominal flap. Utilizing a two-team approach, the recipient site of the left chest was entered from her previous mastectomy incision, along with resection of the surrounding radiated fibrotic skin. A laterally based pectoralis flap was utilized to dissect up to the second rib, which was then partially resected to expose the left internal mammary artery and two veins. Simultaneously, the transversely oriented abdominal flap was elevated by the second surgeon, being particularly careful to preserve both the superior and inferior epigastric vessels on both hemi-abdominal flaps. Dissection proceeded in the prefascial plane above the anterior rectus sheath in a lateral to medial approach. After the favorable right-sided lateral row perforators were identified, the fascia was incised and the rectus muscle exposed, allowing us to trace the vessels down to the origin of the deep inferior epigastric artery and vein in the right lower quadrant. Care was taken to preserve both the superficial and deep inferior epigastric vessels on both sides of the flap during all steps of the dissection. Once the medial portion of the right-sided muscle-sparing TRAM flap was elevated in a MS-2 design, it was found to be quite thin, and so this portion of flap was folded on itself during the harvest. Once the flap was fully islandized, adequate perfusion and bleeding was seen along the entire flap, including zone 4 after the de-epithelization process (Figure 1).

Once the flap was dissected, de-epithelized, and transposed to the left chest, it was folded in half and the following vessels of the tissue were microsurgically anastamosed to those of the left internal mammary system: the superficial inferior epigastric vein in zone 4 of the contralateral fTRAM flap to the medial internal mammary vein, the ipsilateral deep inferior epigastric artery to the internal mammary artery, and the ipsilateral deep inferior epigastric vein to the lateral internal mammary vein. Subsequent additional contouring was performed on the flap and sutured to the inframammary fold of the left chest wall for reinforcement, in lieu of the fibrotic and tenuous local tissue quality. Lastly, the abdominal donor site was closed primarily (2-0 PDS [Ethicon Inc., Somerville, NJ], 3-0 Monocryl [Ethicon Inc.], and 4-0 Monocryl [Ethicon Inc.]) without mesh reinforcement given the minimal tension and healthy quality of her innate tissue. At the conclusion of the case, the flap exemplified excellent color and strong Doppler signals along the entire skin paddle. 

**Figure 2:**
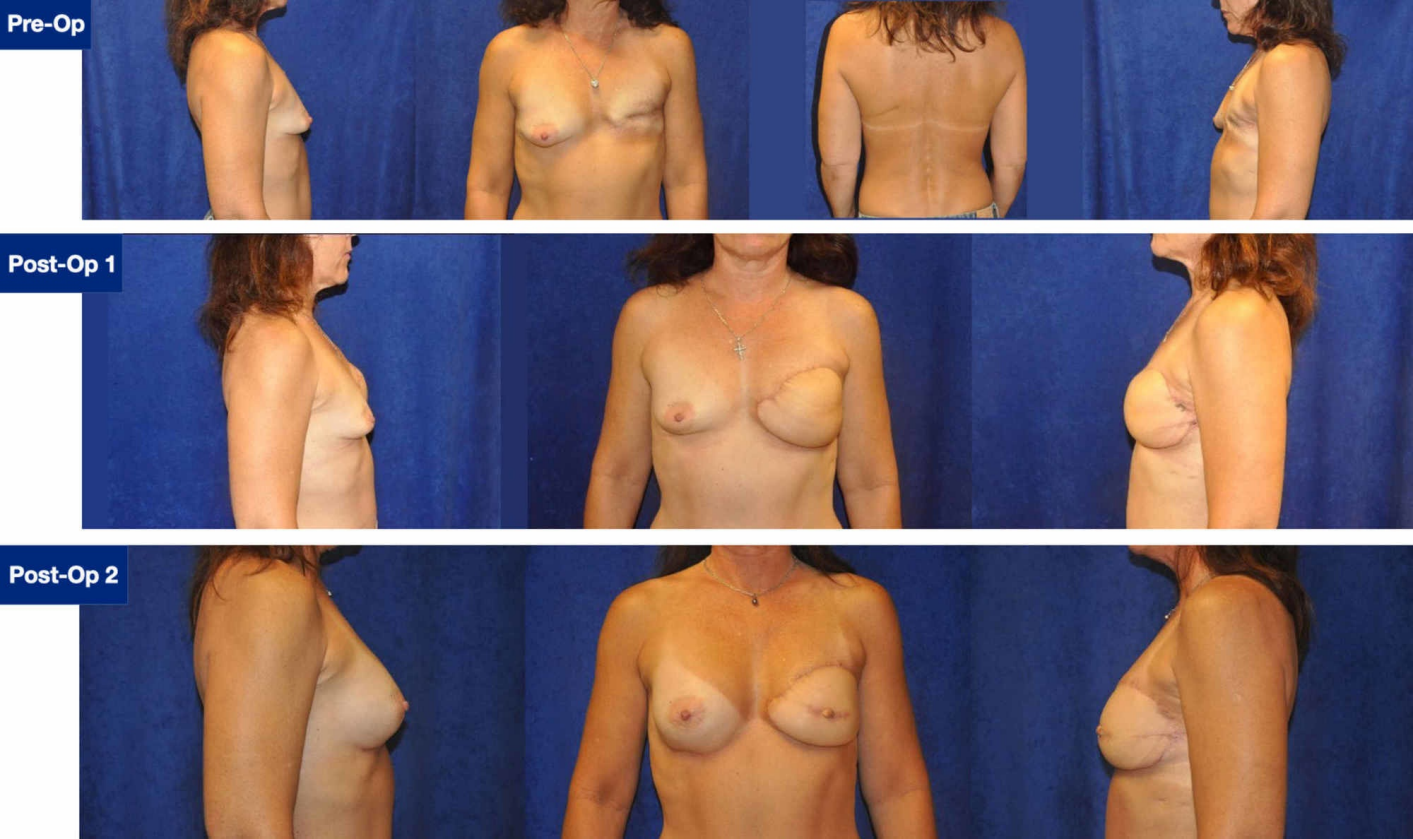
Preoperative presentation and postoperative results. Top: Preoperative photographs of the patient, with a body mass index of 24. Notice the radiated, fibrotic tissue of the left chest, and a prior left posterolateral thoracotomy scar. Middle: Postoperative photographs of the patient, after her initial muscle-sparing supercharged TRAM flap. Bottom: Postoperative photographs of the patient after her symmetrizing right breast augmentation and revision of the left breast with nipple reconstruction. TRAM, transverse rectus abdominis myocutaneous.

Three months after the initial reconstruction, the patient underwent a symmetrizing augmentation of the right breast with a subpectoral silicone implant, and revision of the left breast for improved superior medial fullness and reconstruction of the nipple using a C-Y trilobed flap (Figure 2). The patient expressed that she was highly satisfied with overall symmetry and her final result. 

## Discussion

The traditional pedicle TRAM flap was first described by Hartrampf et al. in 1982, and has become a well-established form of autologous breast reconstruction in postmastectomy breast cancer patients [1]. Since its inception, the TRAM flap has evolved from the pedicle form to the free TRAM flap and finally to the muscle-sparing forms, in order to minimize donor-site morbidity while maximizing flap survival. The fTRAM flap has a blood supply based on the deep inferior epigastric vascular bundle, which has been shown to be the dominant blood supply serving both the ipsilateral skin and variable territories of the contralateral skin paddle through reduced caliber choke vessels, as shown in anatomic cadaveric studies [2].

When the entire fTRAM flap is utilized through a single-sided vascular anastomosis, perfusion to the distal end becomes tenuous and prone to skin or fat necrosis, with a published incidence reaching as high as 32% of patients [3-5]. Given the predictably low perfusion to the distal portions of the fTRAM flap, the majority of surgeons today routinely discard zone 4 and variable portions of zone 3. However, this becomes problematic for surgeons in cases where the entire TRAM flap must be used to cover extensive soft tissue defects. Thus, many variations of the TRAM flap have been designed to provide maximal perfusion to the entire flap.

In 1987, Harashina et al. developed a microvascularly augmented technique in which the pedicled flap was elevated on the superior deep epigastric pedicle along with the contralateral superficial or deep inferior epigastric vessels [6]. The flap was then passed through a subcutaneous tunnel and transposed to the recipient site, where the free pedicle vessels were then microsurgically anastomosed to the axillary vessels, creating a double pedicled TRAM flap with an extended vascular territory [7]. 

In 1991, Beegle introduced the “supercharged” technique, where additional ipsilateral deep inferior epigastric vessels were anastamosed to either the internal mammary or thoracodorsal systems [8,9]. In 2003, Sano et al. showed that the venous supercharging technique can improve TRAM flap survival in a rat model by reducing flap congestion with an additional outflow tract [10].

In alignment with improving flap perfusion and outflow, our team effectively utilized the entire fTRAM flap in a folded fashion, by creating an additional venous anastomosis between the donor superficial inferior epigastric vein of zone 4 and the recipient medial internal mammary vein. This provided our patient with the optimal reconstructive procedure that maximized flap viability while minimizing donor site morbidity. The supercharged fTRAM flap was an excellent autologous reconstructive option in this case due to the necessity of harvesting a large flap in a thin patient with minimal subcutaneous fat.

## Conclusions

Currently, there is a plethora of reconstructive options for both implant and autologous based-breast reconstruction. To date, however, there have not been any studies comparing the supercharged fTRAM with other alternatives, such as the delayed technique or the turbocharged flap, where bilateral deep inferior epigastric pedicles are connected to recipient vessels. Our patient’s experience demonstrates how augmentation of the traditional fTRAM flap with additional blood supply can reduce the likelihood of flap necrosis when the entire fTRAM flap is used, which is often warranted in cases of thin patients presenting with large soft tissue defects. Further comparative studies are, however, necessary to optimize selecting the best reconstructive option for each individual patient.
